# Levonorgestrel Implant Continuation among Postpartum Women Admitted to the Department of Obstetrics and Gynecology of a Tertiary Care Centre

**DOI:** 10.31729/jnma.8254

**Published:** 2023-09-30

**Authors:** Samata Nepal, Shishir Acharya, Anu Marhatta, Sajja Shrestha

**Affiliations:** 1 Department of Community Medicine, Lumbini Medical College, Tansen, Palpa, Nepal; 2 Lumbini Medical College, Tansen, Palpa, Nepal

**Keywords:** *contraception*, *family planning*, *levonorgestrel*, *postpartum period*, *pregnancy*

## Abstract

**Introduction:**

Jadelle (Levonorgestrel) implant is a long-acting reversible contraceptives which is recommended for post-partum contraceptive device due to their high efficacy, convenience, and cost-effectiveness. The continuation of Jadelle implant prevents unintended pregnancies and maintain healthy spacing between the pregnancies, thus improving maternal and child health outcomes. However, Government has endorsed the long-acting reversible contraceptives as immediate postpartum contraception, the status of Jadelle implant continuity is unknown. The aim of this study was to find out the prevalence of levonorgestrel implant continuation among postpartum women admitted to the Department of Obstetrics and Gynecology of a tertiary care centre.

**Methods:**

A descriptive cross-sectional study was conducted among women who had Jadelle implant inserted within 48 hours of delivery admitted to the Department of Obstetrics and Gynecology at a tertiary care centre. Ethical approval was taken from the Institutional Review Committee. Data from 1 July 2020 to 31 December 2020 were collected between 1 July 2022 to 31 December 2022 from the hospital records. Patients were interviewed after 2 years through phone calls. Convenience sampling method was used. The point estimate was calculated at a 95% Confidence Interval.

**Results:**

Out of 157 post-partum women, 145 (92.36%) (88.20-96.52, 95% Confidence Interval) had levonorgestrel implant in-situ.

**Conclusions:**

The prevalence of levonorgestrel implant continuation among postpartum women admitted to the Department of Obstetrics and Gynecology was similar to other studies done in similar settings.

## INTRODUCTION

Immediate post-partum contraception, which involves initiating a long-acting reversible contraceptive (LARC) before discharge from the hospital following delivery, is a best practice method to reduce unintended and short-interval pregnancies. It is more effective at helping women achieve optimal birth intervals when compared to barrier methods.^1^

Despite the high unmet needs for family planning in Nepal, immediate post-partum family planning services, especially LARC, are a critical intervention to increase acceptance without additional healthcare costs.^2^ However, the continuation rates of LARC use among the post-partum population are unclear, which is a vital piece of information for ensuring the effectiveness and sustainability of these interventions.

The aim of the study was to find out the prevalence of levonorgestrel implant continuation among postpartum women admitted to the Department of Obstetrics and Gynecology of a tertiary care centre.

## METHODS

This descriptive cross-sectional study was conducted among women admitted to the Department of Obstetrics and Gynecology who had levonorgestrel implant inserted within 48 hours of delivery at Lumbini Medical College and Teaching Hospital, Tansen, Palpa, Nepal. Data from 1 July 2020 to 31 December 2020 were collected between 1 July 2022 to 31 December 2022 from the hospital records. Ethical clearance was obtained from the Institutional Review Committee (IRC-LMC-29D/021). Post-partum women who had jadelle implant inserted as an immediate post-partum contraceptive method were included in the study. Those who did not give consent were excluded from the study. Convenience sampling method was used. The sample size was calculated by using the following formula:


$$\begin{array}{ll} \text{n} & ={\text{Z}}^{2}\times \frac{\text{p}\times \text{q}}{{\text{e}}^{\text{2}}}\\ & =1.96^{2}\times \frac{0.50\times 0.50}{0.08^{2}}\\ & =151 \end{array}$$

Where,

n = minimum required sample size
Z = 1.96 at 95% Confidence Interval (CI)
p = prevalence taken as 50% for maximum sample size calculation
q = 1-p
e = margin of error, 8%

The minimum sample size required was 151. However, the final sample size taken was 157.

Telephone numbers were obtained from the available records and conducted phone interviews. The purpose and objectives of the study were explained and verbal consent was obtained before the interview. During the phone interviews, the participants were asked about the continuation status of jadelle implant at the end of 2 years, complications experienced so far, the duration of jadelle implant continuation.

Data were entered and analysed using IBM SPSS statistics version 21.0. The point estimate was calculated at 95% CI.

## RESULTS

Out of 157 post-partum women, 145 (92.36%) (88.20-96.52, 95% CI) had levonorgestrel implant in-situ at the end of 2 years. Mean age of participants who continued the jadelle implant was 25.07±5.75 years. More than half of the study participants 77 (53.10%) had received education up to the higher secondary level. A total of 93 (64.13%) of the participants were home makers. Out of 145 participants, 89 (61.38%) had undergone a normal delivery. Among 145 participants, 35 (24.13%) of participants were from the age group of 26-30 years (Table 1).

**Table 1:** Demographic variables of study participants who continued Jadelle implant (n= 145).

Variables	Categories	n (%)
**Age (years)**	<20	34 (23.45)
	21-25	49 (33.79)
	26-30	35 (24.14)
	31-35	22 (15.17)
	>36	5 (3.45)
**Education level**	Primary	26 (17.93)
	Lower secondary	40 (27.59)
	Higher secondary	77 (53.10)
	Bachelor and above	2 (1.38)
**Occupation**	Employed/business	52 (35.86)
	Home maker/no formal occupation	93 (64.14)
Mode of delivery	Normal delivery	89 (61.38)
	Caesarean section delivery	56 (38.62)
Number of pregnancy	1	59 (40.69)
	2	56 (38.62)
	3	20 (13.79)
	4	8 (5.52)
	5	2 (1.38)
Number of living children	1	66 (45.52)
	2	60 (41.38)
	3	18 (12.41)
	4	1 (0.69)

During their antenatal visits, 111 (76.55%) of the participants reported not receiving counselling regarding post-partum contraception (Table 2).

**Table 2:** Variables showing information on counselling (n= 145).

Variables	n (%)
Counseled about immediate post­partum contraception during ANC visits	34 (23.45)
Informed about advantages of Jadelle before insertion	142 (97.93)
Informed about disadvantages of Jadelle before insertion	129 (88.97)

## DISCUSSION

In this study, we found that the immediate postpartum jadelle implantation continuation was 145 (92.40%) at the end of 2 years, which is comparable to studies in other developing countries^3,4^ as well as LARC continuation rates are higher than for other methods.^3^-^5^-^6^ In Nepal the studies regarding the continuity of immediate post-partum Jadelle implant use are limited. One of the reasons for higher continuation rate for jadelle implant is that this is women's choice for post-partum contraception.^7^ jadelle implant is preferred for immediate post-partum contraception due to its shorter duration (5 years) and safety during breastfeeding.^8^ The education level of the women which is about 50% have attained higher secondary education, may be another cause for higher continuation of jadelle. Healthy birth spacing (18-24 months) reduce the risk of anemia, obstetric fistula, precipitous labor, pre-pregnancy obesity and gestational diabetes in maternal health and in infants, pre-term birth, low birth weight and perinatal death may be the adverse outcome.^9-11^

This study had 57.54% of youth (age <25 years) who continued jadelle implant. This finding could not be compared as no other study regarding continuity of jadelle implant as immediate post-partum contraception was found. However, the study done in Tanzania observed young women were more likely to continue immediate post-partum intra-uterine device than older age group.^12^ More than half of the participants in this study had their education level above higher secondary level. Similar findings were observed in other studies too.^5,12^ The uptake of immediate post-partum LARC is higher in the educated women, because they are more aware about appropriate birth interval and avoid unwanted pregnancy.

Our study observed that 61.38% had undergone a normal delivery, who continued the jadelle implant. The study on immediate postpartum implant users in Malawi found that the continuation rates of both levonorgestrel and etonorgestrel implants were high at 2 years, regardless of the method of delivery.^4^ However in another study done in Tanzania, observed that the women delivered by cesarean section had higher continuation rate for LARC when compared to normal vaginal delivery.^12^

In the present study, primi-para woman was 45.51%, followed by the woman with second parity (41.37%), who gave continuity to jadelle implant till two years. The secondary analysis of Nepal Demographic Health Survey 2016 observed parity less than two had influence on use of LARC.^13^ Majority of participants had education level upto higher secondary level, which might have been the influencing factors to desire for next child with proper birth interval and avoidance of unwanted pregnancy.

The American College of Obstetricians and Gynecologists' (ACOG) Committee on Obstetric Practice recommends counseling all women about immediate post-partum contraception initiation.^1^ Our study found that early counseling on the advantages and disadvantages of implant use was associated with a higher jadelle continuation rate. Furthermore, women who do not receive counseling are more likely to discontinue the implant earlier.^14^

Our study has several limitations. The studies regarding continuity of jadelle implant as immediate post-partum contraception could not be retrieved and no published data were available. So, there was difficult in comparing our results. This study is based on the phone interview, so the participants might not have been interested to respond. The chances of recall bias are high for the response regarding antenatal counselling for post-partum contraception. The study only analysed the use of implant and did not consider the use of intra-uterine devices for immediate postpartum contraception. Furthermore, the data were collected exclusively from a single hospital, indicating that the findings cannot be generalized to the entire population.

## CONCLUSIONS

The prevalence of levonorgestrel implant continuation among postpartum women admitted to the Department of Obstetrics and Gynecology was similar to other studies done in similar settings. Immediate postpartum jadelle insertion has a high continuation rate and well-accepted and tolerated when inserted immediately postpartum before hospital discharge. It should be made widely available to improve postpartum contraceptive coverage in Nepal. Additional studies are needed to assess continuation rates across diverse settings in the country.
